# RNA Based Approaches to Profile Oncogenic Pathways From Low Quantity Samples to Drive Precision Oncology Strategies

**DOI:** 10.3389/fgene.2020.598118

**Published:** 2021-02-05

**Authors:** Anja van de Stolpe, Wim Verhaegh, Jean-Yves Blay, Cynthia X. Ma, Patrick Pauwels, Mark Pegram, Hans Prenen, Dirk De Ruysscher, Nabil F. Saba, Susan F. Slovin, Karen Willard-Gallo, Hatim Husain

**Affiliations:** ^1^Philips MPDx, Eindhoven, Netherlands; ^2^Philips Research, Eindhoven, Netherlands; ^3^Medical Oncology, Université Claude Bernard Lyon 1, Lyon, France; ^4^Centre Léon Bérard, Lyon, France; ^5^Medicine, Division of Oncology, Section of Medical Oncology, Washington University School of Medicine, St. Louis, MO, United States; ^6^Molecular Pathology, Centre for Oncological Research (CORE), University of Antwerp, Antwerp, Belgium; ^7^Stanford University School of Medicine, Clinical Research, Stanford Cancer Institute, Stanford, CA, United States; ^8^Oncology Department, Head of Phase I – Early Clinical Trials Unit, Clinical Trial Management Program, Oncology Department, Antwerp University Hospital, Antwerp, Belgium; ^9^Oncology-Radiotherapy, Maastro/Maastricht University Medical Center, Maastricht, Netherlands; ^10^Department of Hematology and Medical Oncology, Emory University School of Medicine, Atlanta, GA, United States; ^11^Department of Otolaryngology, Emory University School of Medicine, Atlanta, GA, United States; ^12^Head and Neck Medical Oncology Program, Winship Cancer Institute of Emory University, Atlanta, GA, United States; ^13^Department of Medicine, MSKCC, New York, NY, United States; ^14^Molecular Immunology Laboratory, Institut Jules Bordet, Brussels, Belgium; ^15^University of California, San Diego, La Jolla, CA, United States

**Keywords:** oncology precision medicine, treatment prediction, signaling pathway activity, mRNA profiling, low input analytes

## Abstract

Precision treatment of cancer requires knowledge on active tumor driving signal transduction pathways to select the optimal effective targeted treatment. Currently only a subset of patients derive clinical benefit from mutation based targeted treatment, due to intrinsic and acquired drug resistance mechanisms. Phenotypic assays to identify the tumor driving pathway based on protein analysis are difficult to multiplex on routine pathology samples. In contrast, the transcriptome contains information on signaling pathway activity and can complement genomic analyses. Here we present the validation and clinical application of a new knowledge-based mRNA-based diagnostic assay platform (OncoSignal) for measuring activity of relevant signaling pathways simultaneously and quantitatively with high resolution in tissue samples and circulating tumor cells, specifically with very small specimen quantities. The approach uses mRNA levels of a pathway’s direct target genes, selected based on literature for multiple proof points, and used as evidence that a pathway is functionally activated. Using these validated target genes, a Bayesian network model has been built and calibrated on mRNA measurements of samples with known pathway status, which is used next to calculate a pathway activity score on individual test samples. Translation to RT-qPCR assays enables broad clinical diagnostic applications, including small analytes. A large number of cancer samples have been analyzed across a variety of cancer histologies and benchmarked across normal controls. Assays have been used to characterize cell types in the cancer cell microenvironment, including immune cells in which activated and immunotolerant states can be distinguished. Results support the expectation that the assays provide information on cancer driving signaling pathways which is difficult to derive from next generation DNA sequencing analysis. Current clinical oncology applications have been complementary to genomic mutation analysis to improve precision medicine: (1) prediction of response and resistance to various therapies, especially targeted therapy and immunotherapy; (2) assessment and monitoring of therapy efficacy; (3) prediction of invasive cancer cell behavior and prognosis; (4) measurement of circulating tumor cells. Preclinical oncology applications lie in a better understanding of cancer behavior across cancer types, and in development of a pathophysiology-based cancer classification for development of novel therapies and precision medicine.

## Introduction

Cellular mechanisms of cancer can be described in terms of abnormal activity of a discrete number of signal transduction pathways that control crucial cellular functions and play important roles in both physiology (e.g., embryonic development, immune response) and pathophysiology. They can be categorized as hormone driven pathways [e.g., estrogen receptor (ER), androgen receptor (AR), progesterone receptor (PR), and glucocorticoid receptor (GR) pathways], growth factor pathways (e.g., PI3K, MAPK-AP1, JAK-STAT1/2 and JAK-STAT3), the inflammatory pathway (NFκB), and developmental pathways [e.g., Wnt, Hedgehog (HH), TGFβ, and Notch pathways].

Signal transduction pathways drive tumor growth and metastasis, either as a single active pathway (e.g., the ER pathway in luminal A breast cancer) or through cooperation or crosstalk between signaling pathways (e.g., the MAPK and TGFβ pathways) (Sundqvist et al., 2013). Abnormal pathways are at the core of cancer pathophysiology and are frequently caused by molecular aberrations in the cancer genome in interaction with the cancer cell microenvironment (Hanahan and Weinberg, 2011; Bose et al., 2013). Signaling pathway activity can either be increased to drive growth or metastasis of a tumor, as is frequently the case for the PI3K pathway, or decreased in case of a signaling pathway that normally serves as a tumor suppressor. Some pathways, such as the TGFβ pathway, can exert tumor promoting and suppressive effects depending on the cancer type. A drug can either target the signal transduction pathway at the receptor, for example trastuzumab to target the HER2 receptor, or at downstream nodes, for example a PI3K inhibitor to target the PIK3CA signaling molecule or everolimus targeting the mTOR protein (Baudino, 2015; Sieuwerts et al., 2020). Many targeted drugs, such as those which target the ER, PI3K and MAPK signaling pathways, have been clinically approved based on evidence of clinical benefit and are being used to treat various cancer types (Massard et al., 2017; Dugger et al., 2018; Marquart et al., 2018; National Cancer Institute, 2021). Emerging evidence suggests that activity of specific signaling pathways are involved in response and resistance to a variety of therapies including targeted therapy, immunotherapy, and chemotherapy.

## A Major Challenge in Oncology Diagnostics and Treatment Decisions: Identification of the Tumor Driving Signaling Pathway Activity in Cancer

Traditionally, the selection of targeted drugs has been based in immunohistochemistry (IHC, i.e., staining for ER, PR and HER2 protein expression in breast cancer) and can be applied to formalin fixed paraffin embedded (FFPE) tissue samples. IHC staining is currently semi-quantitative, however, and multiplexing staining remains a major challenge. Correct normalization of staining signals can be inconsistent, and sample preparation and staining protocols can vary substantially among pathology departments. Expression levels of signaling proteins do not provide information on their functional activity and activity of the associated signaling pathway (Hemert et al., 2020). The presence of ER in the cell nucleus is required for ER pathway activity, but ER expression alone is not sufficient as the estrogen ligand is necessary to activate ER and consequently the ER signaling pathway (Katzenellenbogen et al., 1993; Hemert et al., 2020). This explains the variations in ER pathway activity in an evenly distributed ER positive tumor (Yang et al., 2019; Inda et al., 2020). The functional state of signaling proteins can be determined by complex and dynamic alterations of the protein structure, which require quantitative measurement of a variety of post-translational modifications which affect protein activity. Staining methods using antibodies that recognize a specific phosphorylation state of a protein have been investigated extensively, but are not always sufficiently reliable (Mandell, 2008). Mass spectrometry proteomics approaches can be used to measure post-translational modifications of a range of receptor, signaling and transcription factor proteins (Mnatsakanyan et al., 2018), but diagnostic applications can be complicated since the analysis is limited to fresh frozen (FF) tissue samples and requires a relatively large amount of tissue to obtain a reliable result. An alternative means to measure activity of a signaling protein is to provide a substrate to the protein to measure enzymatic activity, but this method is similarly limited to fresh frozen, non-denatured samples (Shi et al., 2018). Directly measuring transcription factor activity is even more complex and requires fresh cells or tissue (Browning et al., 2009). Protein analysis approaches have been of limited help to measure the activation state of a signaling pathway and as a method to predict therapy response.

The availability of Next Generation Sequencing (NGS) techniques has enabled identifying tumor-driving gene mutations in many histologies of cancer. This has resulted in a number of therapeutic successes, as evident from FDA approved companion diagnostic assays based on identification of a specific gene mutation to predict therapy response (FDA, 2020). FDA approval was recently obtained for the use of the PI3K inhibitor drug alpelisib in breast cancer patients with a *PIK3CA* mutation, based on the results of the SOLAR-1 trial (André et al., 2019). Other mutations may be clinically used independent of FDA approval, for example activating mutations in the *ESR1* gene coding for the estrogen receptor and associated with resistance to hormonal therapy (Jeselsohn et al., 2015). For many mutations in signaling pathway-related genes, for example in the Notch genes, it remains hard to establish the clinical relevance of the mutation. A clear link between the mutation and activity of the corresponding signaling pathway can often not be made reliably (Janku, 2017; van de Stolpe, 2019; Martin et al., 2020). An important factor, partially explaining this disappointing result, is that the cancer cell phenotype is determined not only by genomic mutations, but also by epigenetic dysregulations and by interactions with cells in the tumor microenvironment, e.g., fibroblasts and a variety of immune cells (Hanahan and Weinberg, 2011). This makes it essential to measure functional signaling pathway activity in a cancer tissue sample, while taking into account the challenge to distinguish between the phenotypes of cancer cells and cell types in the microenvironment.

## Measuring Phenotypic Signaling Pathway Activity in a Cancer Tissue Sample From mRNA Expression

RNA-based methods to measure signaling pathway activity have advanced during the past decade to provide information on signaling pathway activity based on mRNA expression data of a tumor sample. However, using mRNA expression levels as a proxy for the presence of corresponding activated signaling molecules or transcription factors can be inaccurate. First, mRNA expression levels are unreliable indicators of corresponding protein levels because their production and turnover rates are frequently not matched. Second, signaling protein levels are in general not related to the functional activity state of the protein, which is determined by post-translational modifications, such as specific phosphorylations. Nevertheless, mRNA expression levels can provide information on activity of the transcription factors that produce them, and indirectly on activity of signaling pathways that lead to activation of such transcription factors.

A number of RNA-based pathway analysis tools are available, such as Ingenuity Pathway Analysis (Qiagen), Gene Set Enrichment Analysis (GSEA) (Subramanian et al., 2005) and DAVID (Huang et al., 2009). They use pathway information from databases such as KEGG^1^, and WikiPathways^2^. The term “pathways” there usually refers to a variety of intracellular molecular mechanisms, not specifically to activity of signal transduction pathways (Subramanian et al., 2005; Huang et al., 2009). These tools are mostly used to discover which “pathways” differ between two groups of samples, by looking at the differentially expressed genes between the two groups, and identifying the “pathways” in which these differentially expressed genes are overrepresented. A drawback of such methods is that typically analysis starts unbiased, with thousands of “pathways” being evaluated for their difference between the two groups of samples. On the one hand, such a discovery setup may be limited by testing corrections, while many findings may emerge that are not reflections of the tumor driving pathway. For instance, when comparing normal tissue to cancer tissue, “pathways” involved in cell cycling may appear, and can signify an effect rather than a driver. In addition, pathways known to drive certain tumors in some cases may not be identified or identified incorrectly. A GSEA experiment analyzing canonical pathways between 32 colon adenoma vs. 32 normal colon samples from GSE8671, shown in Table 1, failed to identify the Wnt pathway as being upregulated in colon adenomas, while it is known to be the first activated pathway in practically all adenomas. The first occurrence of a Wnt pathway was on position 257 of the result list, with a corrected *p*-value of 0.91. Some Wnt pathways were reported to be up-regulated in the normal samples instead of the adenomas. A major cause of such findings is the inclusion of genes encoding for signaling proteins in “pathways,” which are only very loosely coupled to protein levels and activation.

**TABLE 1:** Results of a Gene Set Enrichment Analysis on 32 colon adenomas vs. 32 normal colon samples from GSE8671, using 1,502 canonical pathways from the curated pathway database from MSigDB (c2/cp).

Up in	Position	Name	nom.p	fdr.q	fwer.q
**A**					
Adenoma	1	PID_FOXM1_PATHWAY	0.000	0.224	0.133
Adenoma	2	PID_AURORA_A_PATHWAY	0.000	0.211	0.222
Adenoma	3	REACTOME_CHROMOSOME_MAINTENANCE	0.000	0.193	0.262
Adenoma	4	KEGG_CELL_CYCLE	0.002	0.151	0.271
Adenoma	5	REACTOME_G2_M_CHECKPOINTS	0.002	0.141	0.303
Adenoma	6	REACTOME_CELLULAR_RESPONSE_TO_HEAT_STRESS	0.002	0.121	0.306
Adenoma	7	REACTOME_REGULATION_OF_TP53_ACTIVITY_THROUGH_PHOSPHORYLATION	0.000	0.105	0.312
Adenoma	8	PID_MYC_ACTIV_PATHWAY	0.000	0.093	0.314
Adenoma	9	REACTOME_CELL_CYCLE_CHECKPOINTS	0.002	0.083	0.314
Adenoma	10	REACTOME_REGULATION_OF_HSF1_MEDIATED_HEAT_SHOCK_RESPONSE	0.002	0.088	0.352
Adenoma	11	REACTOME_BASE_EXCISION_REPAIR	0.000	0.085	0.364
Adenoma	12	BIOCARTA_G2_PATHWAY	0.000	0.081	0.374
Adenoma	13	REACTOME_DNA_REPAIR	0.000	0.079	0.384
Adenoma	14	REACTOME_RHO_GTPASES_ACTIVATE_FORMINS	0.014	0.077	0.397
Adenoma	15	REACTOME_METABOLISM_OF_NUCLEOTIDES	0.000	0.072	0.397
Adenoma	16	REACTOME_REGULATION_OF_RAS_BY_GAPS	0.002	0.068	0.403
Adenoma	17	REACTOME_PROTEIN_FOLDING	0.002	0.065	0.409
Adenoma	18	SA_G1_AND_S_PHASES	0.002	0.063	0.412
Adenoma	19	REACTOME_TELOMERE_MAINTENANCE	0.000	0.061	0.419
Adenoma	20	REACTOME_TP53_REGULATES_TRANSCRIPTION_OF_GENES_INVOLVED_IN_G2_CELL_CYCLE_ARREST	0.000	0.059	0.421
**B**					
Adenoma	125	REACTOME_DEGRADATION_OF_BETA_CATENIN_BY_THE_DESTRUCTION_COMPLEX	0.018	0.036	0.724
Adenoma	257	REACTOME_TCF_DEPENDENT_SIGNALING_IN_RESPONSE_TO_WNT	0.015	0.046	0.914
Adenoma	387	REACTOME_SIGNALING_BY_WNT	0.055	0.179	1.000
Adenoma	482	PID_BETA_CATENIN_DEG_PATHWAY	0.227	0.363	1.000
Adenoma	496	PID_BETA_CATENIN_NUC_PATHWAY	0.244	0.393	1.000
Adenoma	507	REACTOME_FORMATION_OF_THE_BETA_CATENIN_TCF_TRANSACTIVATING_COMPLEX	0.324	0.420	1.000
Adenoma	540	WNT_SIGNALING	0.330	0.463	1.000
Adenoma	546	KEGG_WNT_SIGNALING_PATHWAY	0.334	0.475	1.000
Adenoma	561	REACTOME_DEACTIVATION_OF_THE_BETA_CATENIN_TRANSACTIVATING_COMPLEX	0.405	0.483	1.000
Adenoma	568	BIOCARTA_WNT_PATHWAY	0.425	0.487	1.000
Adenoma	652	REACTOME_SIGNALING_BY_WNT_IN_CANCER	0.792	0.764	1.000
**C**					
Normal	1	KEGG_NITROGEN_METABOLISM	0.000	0.178	0.095
Normal	2	KEGG_ALDOSTERONE_REGULATED_SODIUM_REABSORPTION	0.000	0.242	0.209
Normal	3	KEGG_PROXIMAL_TUBULE_BICARBONATE_RECLAMATION	0.006	0.216	0.263
Normal	4	KEGG_LEUKOCYTE_TRANSENDOTHELIAL_MIGRATION	0.002	0.260	0.351
Normal	5	REACTOME_NITRIC_OXIDE_STIMULATES_GUANYLATE_CYCLASE	0.000	0.241	0.380
Normal	6	REACTOME_PLATELET_HOMEOSTASIS	0.000	0.206	0.383
Normal	7	KEGG_VASCULAR_SMOOTH_MUSCLE_CONTRACTION	0.000	0.183	0.391
Normal	8	REACTOME_DIGESTION	0.000	0.181	0.412
Normal	9	REACTOME_GLUCAGON_LIKE_PEPTIDE_1_GLP1_REGULATES_INSULIN_SECRETION	0.000	0.163	0.415
Normal	10	REACTOME_DIGESTION_AND_ABSORPTION	0.000	0.148	0.418
Normal	11	REACTOME_GLUCAGON_SIGNALING_IN_METABOLIC_REGULATION	0.000	0.147	0.433
Normal	12	REACTOME_SUMOYLATION_OF_INTRACELLULAR_RECEPTORS	0.000	0.144	0.444
Normal	13	REACTOME_FCGR3A_MEDIATED_IL10_SYNTHESIS	0.004	0.137	0.451
Normal	14	REACTOME_AQUAPORIN_MEDIATED_TRANSPORT	0.000	0.131	0.455
Normal	15	REACTOME_STIMULI_SENSING_CHANNELS	0.000	0.131	0.470
Normal	16	REACTOME_FOXO_MEDIATED_TRANSCRIPTION	0.004	0.140	0.495
Normal	17	REACTOME_RHO_GTPASES_ACTIVATE_PAKS	0.002	0.151	0.533
Normal	18	NABA_ECM_GLYCOPROTEINS	0.006	0.154	0.547
Normal	19	REACTOME_BINDING_AND_UPTAKE_OF_LIGANDS_BY_SCAVENGER_RECEPTORS	0.002	0.152	0.558
Normal	20	REACTOME_CREATION_OF_C4_AND_C2_ACTIVATORS	0.006	0.151	0.573
**D**					
Normal	406	PID_WNT_CANONICAL_PATHWAY	0.200	0.401	1.000
Normal	672	PID_WNT_SIGNALING_PATHWAY	0.534	0.619	1.000
Normal	783	REACTOME_BETA_CATENIN_PHOSPHORYLATION_CASCADE	0.777	0.804	1.000
Normal	791	REACTOME_WNT_LIGAND_BIOGENESIS_AND_TRAFFICKING	0.852	0.837	1.000
Normal	792	REACTOME_WNT5A_DEPENDENT_INTERNALIZATION_OF_FZD4	0.829	0.836	1.000

***(A)** 20 most upregulated pathways in colonadenomas. **(B)** Canonical Wnt-related pathways upregulated in colon adenomas. **(C)** 20 most upregulated pathways in normal colon samples. **(D)** Canonical Wnt-related pathways upregulated in normal colon samples. Per pathway the rank is given in the list of upregulated pathways (position), the nominal p-value (nom.p), false-discovery-rate corrected p-value (fdr.q), and the family-wise error corrected p-value (fwer.q).*

Even if above pathway analysis approaches yield relevant “pathways” that are differentially expressed, then a step is to be made to translate them into a gene expression profile or a classifier that can be applied on an individual test sample. This requires a training step on samples with known ground truth pathway status. Since many of these “pathway” gene sets have been derived in a data-driven manner, one should be cautious when applying them on test samples of a different nature than the original discovery set. Data-driven discovery approaches are difficult to properly assess functional activity of tumor-driving pathways. Here we will present a novel approach that focuses on specific pathway activations that are distinct from traditional gene enrichment set analysis.

## Methods: Design of a New Highly Sensitive Assay Platform to Quantitatively Measure Activity of Signal Transduction Pathways Across Cell and Tissue Types and Validation

Starting from the opportunities provided by the transcriptome, we have developed an assay (OncoSignal) to quantitatively measure activity of signal transduction pathways across cell and tissue types. At the core of the approach is a mathematical Bayesian model which quantitatively infers activity of signal transduction pathways from mRNA measurements of a small number of selected direct target genes of the respective signaling pathway-associated transcription factor (Verhaegh and van de Stolpe, 2014; Verhaegh et al., 2014; van Ooijen et al., 2018; van de Stolpe, 2019; van de Stolpe et al., 2019a). By selecting for high evidence direct target genes of the transcription factor, this approach builds on knowledge obtained on mechanisms of signal transduction and gene transcription regulation (van Hartskamp et al., 2019).

### A Bayesian Model of Target Gene Activation

**FIGURE 1:**
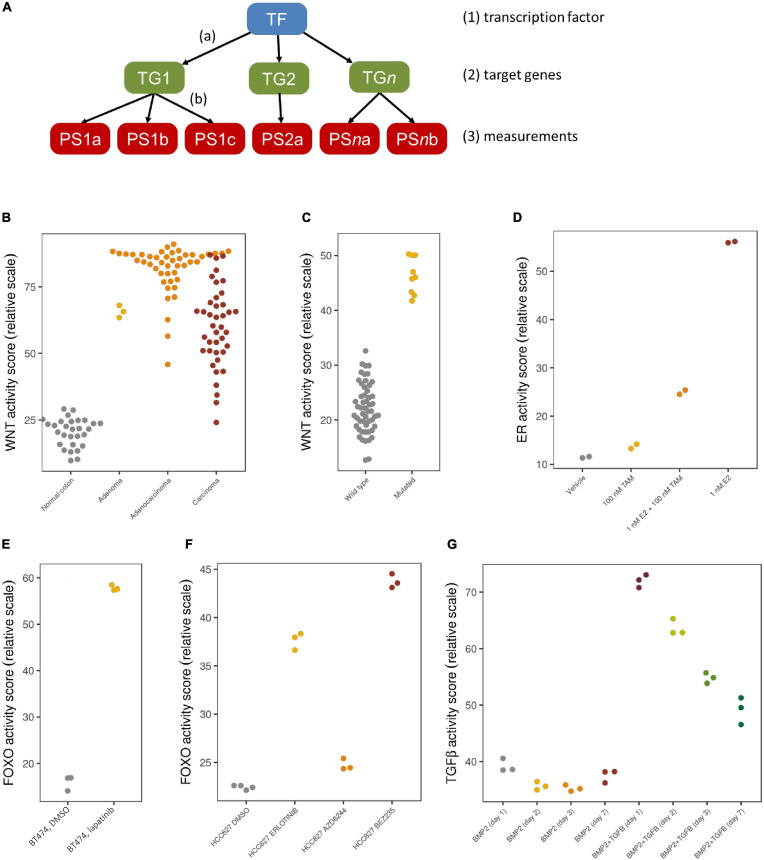
Bayesian model and validation results. **(A)** The network structure of the Bayesian pathway model, with causal, probabilistic dependencies. **(B–G)** Validation results with pathway activity scores represented on a 0–100 scale; each dot is an individual sample. **(B)** Normal colon samples (gray) show a low Wnt pathway activity, while colon adenomas (yellow), adenocarcinomas (orange) and carcinomas (red) show a high Wnt pathway activity; microarray data from GSE20916. **(C)** Medulloblastoma samples with a driving *CTNNB1* mutation (yellow) show a high Wnt activity, while other medulloblastoma samples (gray) show a low Wnt activity; microarray data from GSE10327. **(D)** ER pathway activity in MCF7 cell lines is low if treated with vehicle (gray) or only tamoxifen (yellow), high if treated with E2 (red), which is reduced again after adding tamoxifen (orange); microarray data from GSE53734. **(E)** FOXO activity is low in control BT474 cell lines (gray), indicating a high PI3K activity, but FOXO is high again (and PI3K low) after treatment with lapatinib (yellow); microarray data from GSE16179. **(F)** Quantitative differences in FOXO activity in HCC827 cell lines treated with DMSO (gray), erlotinib (yellow), AZD6244 (orange) and BEZ235 (red); microarray data from GSE51212. **(G)** Quantitative differences over the course of time (days 1, 2, 3, 7) without (first four groups) and with TGFβ stimulation (last four groups); microarray data from GSE84500.

We have employed a Bayesian network model that describes the causal relation between transcription factor activation of a certain pathway and the mRNA expression levels of the corresponding direct target genes (Verhaegh et al., 2014), as shown in Figure 1A. The model contains three types of nodes: (1) a transcription factor complex (TF), (2) high evidence direct target genes (TG), and (3) measurement nodes representing the corresponding probes or probe sets (PS) of each of the target genes. Based on an elaborate literature study, we selected genes as direct target genes if they were supported by multiple types of evidence, such as motif analysis on the transcription factor, its actual binding to promotor regions (e.g., ChIP-seq experiments), and differential expression experiments, and supported by ongoing clinical trials. By choosing direct target genes, we ensure maximum specificity of the models. Furthermore, this reduces dependency on specific tissue-dependent contexts, making that the models can be used across different tissue types.

Given the network structure, we subsequently define parameters describing (a) the relation between the transcription factor complex and the direct target genes, and (b) the relation between each target gene and its corresponding measurements. The parameters of the first layer (a) are based on literature insights, whereas those of the second layer (b) are calibrated based on measurements on samples with a known ground truth status for activity of the respective signal transduction pathway, from which typical measurement levels are deduced in an inactive or an active state. These ground truth samples may come from patient studies, such as for the Wnt pathway, which is known to be activated in colon adenomas and inactive in normal colon samples, or from controlled cell line experiments in which a default inactive pathway is activated by adding ligands, or a constitutively activated pathway is blocked by means of blocking compounds.

### Biological Validation

After building and calibrating Bayesian pathway models as described above, the models can be applied on subsequent individual test samples by taking such a sample’s measurements and feeding them into the model after which Bayesian inference is used to re-engineer the odds that the transcription factor must have been activated compared to those that are not activated. These odds are represented on a log2 scale, with a positive value indicating that there is more evidence for an active pathway, and a negative value indicating more evidence of an inactive pathway. For ease of comparison, these log2 values are further normalized to a 0–100 scale.

We have developed Bayesian network models to assess the phenotypic activity of the PI3K, MAPK-AP1, JAK-STAT1/2 and JAK-STAT3, NFκB, ER, AR, Notch, TGFβ, Wnt and HH signaling pathways using Affymetrix expression microarray data (HG-U133Plus2.0 arrays), and we tested each of them extensively on data sets from publicly available sources such as the Gene Expression Omnibus to show that they give a correct read-out of activity in samples with known ground truth pathway status (Verhaegh et al., 2014; Creemers et al., 2018; van de Stolpe et al., 2018; van Ooijen et al., 2018; Bouwman et al., 2020; Canté-Barrett et al., 2020; Inda et al., 2020). Some validation data for Wnt, ER, FOXO, and TGFβ is shown in Figures 1B–G, illustrating that the tests accurately indicate pathway activity known from disease etiology (1B) or mutation information (1C), and represent changes in activity due to treatment (1D and 1E). Pathway activity is assessed not only in a qualitative manner, but differences in effect from various treatment compounds or time series can even be observed quantitatively (1F and 1G).

The signal transduction pathway (STP) assays have been biologically validated on a variety of cell types and are in principle applicable to all healthy and diseased cell and tissue types. The major reason for this broad applicability is the focus on transcription factor target genes. This maximally eliminates influences of cell type-specific proteins on the target gene levels. Another feature of the Bayesian computational network model is that it can deal well with variability in input data, including conflicting data such as target genes that are not expressed in a specific sample despite the transcription factor being active, or target genes that are expressed despite an inactive transcription factor. Third, target genes were not selected based on relevance for a specific disease or tissue type, but solely as reliable readout for transcription factor activity. When expression levels of individual pathway target genes vary between samples and between different cell types, the Bayesian reasoning principle allows robust interpretation of these mRNA levels. This explains why the STP assays can deliver reliable pathway activity measurements across patient samples in spite of the variation that is inherent to such samples. The same signal transduction pathways that play a role in cancer, also determine activity or immunosuppression of the many cell types that together generate the innate and adaptive immune response, and as a consequence STP tests are being studied to measure activity of the immune system (van de Stolpe et al., 2019b).

### Pathway Assays for Routine Clinical Samples

Our STP assays were first built and tested on Affymetrix expression microarray data, having the advantage of access to publicly available datasets from the GEO database, enabling model validation on multiple independent preclinical and clinical datasets (GEO database, 2021). To facilitate clinical use and enable assay performance on FFPE tissue samples, assays for MAPK-AP1, PI3K, ER, AR, Notch, TGFβ, and HH pathways have recently been adapted to RT-qPCR measurements of mRNA. Additional advantages of qPCR-based tests are “in house” use on regular PCR equipment for a short time-to-result (typically within 3 h) and use on small or low quality FFPE samples.

To build these RT-qPCR pathway assays, we first reduced the number of target genes used per pathway to around 12. This still enables robust behavior of the assays, while maintaining specificity, and all validation results were confirmed with these reduced gene lists. Next, we used the very same calibration samples to determine the model parameters with the only difference that we measured them again with RT-qPCR. In this way, we ensure compatibility between the microarray-based and RT-qPCR based STP assays.

### Comparing Pathway Scores to Reference Distributions

**FIGURE 2:**
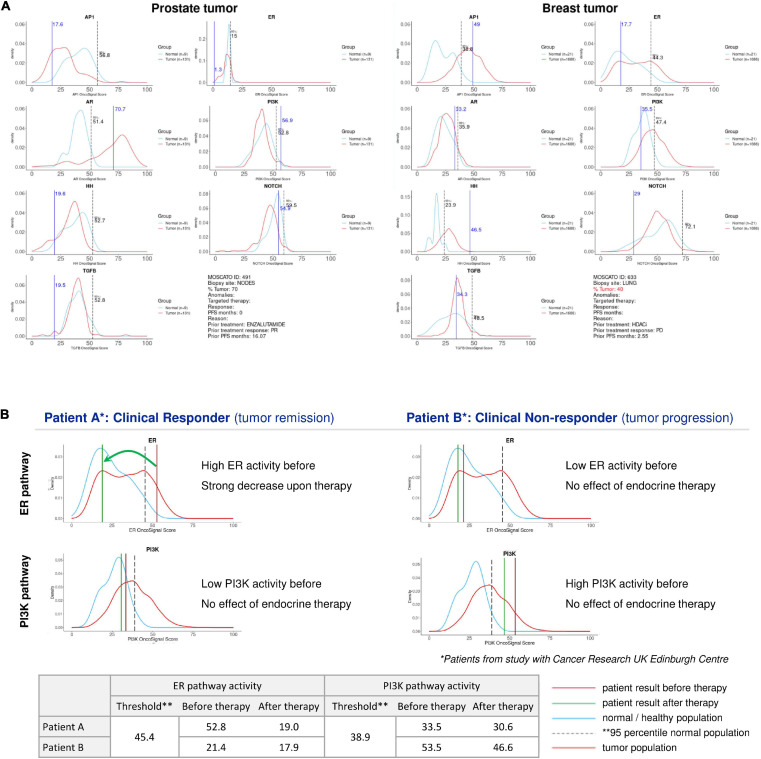
Patient report for signaling pathway analysis with OncoSignal pathway activity tests. Visualized are for each signaling pathway the pathway activity distribution in healthy reference tissue (blue line) and in primary cancers originating from this tissue (prostate and breast) (red line); the dotted vertical line indicates the 95% confidence interval of normal pathway activity; the vertical blue line indicates the measured pathway activity in the analyzed patient tissue sample. If the blue line is located outside the (right) 95th percentile of normal, pathway activity is considered potentially tumor driving and targetable. **(A)** Examples of two individual patient samples from the EIT-PACMAN study (Martin et al., 2020), in which samples were analyzed from hard-to-treat patients from the MOSCATO study (Massard et al., 2017). For each patient pathway activity scores are shown for the ER, AR, PI3K, Hedgehog, MAPK-AP1, Notch, TGFβ pathways. Left: prostate cancer patient; right: breast cancer patient. For the prostate cancer patient (vertical line) the AR pathway activity score exceeded the 95th percentile of normal, while for prostate cancer in general AR pathway activity also exceeds the normal threshold (red line); consequently, the AR pathway was defined as a potentially targetable tumor driving pathway for this patient. Similarly, the ER pathway was abnormally active in the advanced breast cancer patient, providing a treatment target. **(B)** Example of two individual patient samples from Inda et al. (2020). For two patients with an ER positive breast cancer, ER (top) and PI3K (bottom) pathway activity scores are shown before (brown vertical line) and after (green vertical line) neoadjuvant hormonal aromatase inhibitor therapy. The left patient is a responder to hormonal therapy, and the increased ER pathway activity before therapy returned to normal during therapy. The right patient is a non-responder to hormonal therapy, and instead of the ER pathway, the PI3K pathway is the most likely tumor driving pathway. Below, the actual measured pathway activity scores are depicted.

When performing an STP analysis on an individual patient sample to help guide the decision on targeted therapy, it will be necessary to know whether and to which extent a specific signaling pathway activity is considered abnormal, and if increased, whether it is likely to be tumor driving and targetable. This requires the definition of a normal pathway activity range including a pathway activity threshold. To obtain this, STP activity analysis was performed on healthy tissue samples, and the 95th percentile of normal was defined as threshold above which the signaling pathway activity in tumor tissue is considered to be abnormal (Martin et al., 2020). When the pathway activity score on an individual sample is defined as increased, it can be considered as a potential tumor driving signaling pathway. To increase the likelihood that the identified pathway is indeed tumor driving, it is of value to know whether this pathway is more often activated in the analyzed cancer type. For this purpose, sets of cancer tissue samples have been compared with the reference pathway activity range for different cancer types, and frequently activated signaling pathways were defined as likely tumor driving pathways for the respective cancer types. To illustrate this, for the ER and AR pathways, the average pathway activity score was observed abnormally high in luminal breast cancer and primary prostate cancer, respectively, indicating a role as tumor driving pathway in these cancer types (Verhaegh et al., 2014; van de Stolpe et al., 2019a; Inda et al., 2020). If an abnormal ER pathway activity is measured in an ER positive breast cancer sample, this can be considered as a tumor driving pathway and can be designated as a therapy target, and it may be inferred that a patient can be a good candidate for hormonal therapy (see Figure 2B, left). If despite an ER positive IHC result, the ER pathway appears to be not abnormally active, the patient may have another tumor driving pathway active, for example the PI3K pathway (Figure 2B, right) which may be of importance for therapy choice. Once the normal reference values are known for a specific tissue type, for each patient sample a patient report can be produced containing information for all analyzed signaling pathways (Figure 2A) and evaluated as potential targets.

## Results: Application of Findings

### The Advantage of Adding RNA-Based Information on Signaling Pathway Activity to DNA-Based Genomic Mutation Analysis

**FIGURE 3:**
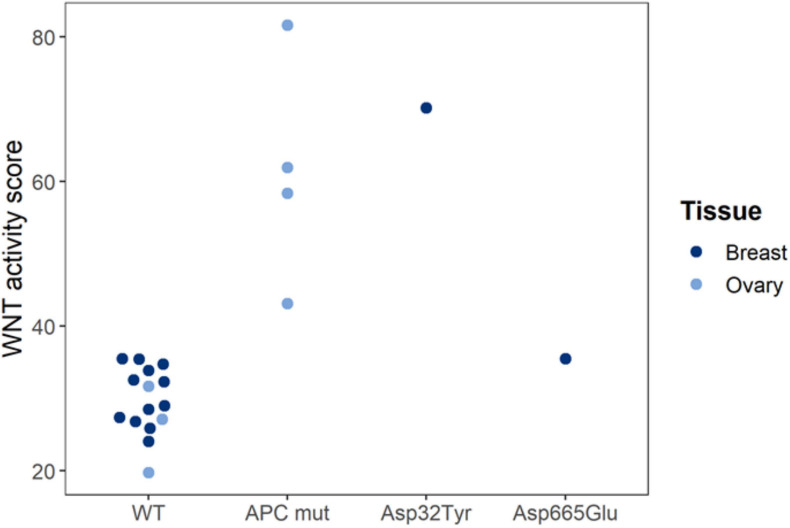
Functional characterization of Wnt pathway mutations in PDX mice. Wnt pathway activity scores were measured in tissue samples from human ovarian and breast cancer PDX (xenograft) mice. Loss of APC and gain of β-catenin (*CTNNB1* gene) protein function are known to result in increased Wnt pathway activity. Wnt pathway activity scores were normal in the reference (WT), increased in loss-of-function APC-mutated ovary cancer and in gain-of-function *CTNNB1* Asp32Tyr mutated breast cancer, and normal in non-functionally mutated *CTNNB1* Asp665Glu breast cancer. Pathway tests were adapted for use in mouse PDX models (to exclude interference of mouse model microenvironment). Pathway Activity Scores presented on a 0–100 scale. Tissue samples were analyzed in a collaboration with Charles River Labs (CRL) (Verhaegh et al., 2018).

Our findings have shown that STP analysis of a cancer tissue sample may assist in functionally characterizing a gene mutation; for example, an unknown mutation in a gene encoding a signaling protein is more likely to be a functional mutation if the corresponding pathway was found active (Figure 3). We show that Wnt pathway activity scores were measured in tissue samples from human ovarian and breast cancer PDX (xenograft) mice. Loss of APC and gain of β-catenin (*CTNNB1* gene) protein function are known to result in increased Wnt pathway activity. Wnt pathway activity scores were normal in the reference (WT), increased in loss-of-function APC-mutated ovary cancer and in gain-of-function *CTNNB1* Asp32Tyr mutated breast cancer, and normal in non-functionally mutated *CTNNB1* Asp665Glu breast cancer (Figure 3).

STP activity analysis is also expected to facilitate the search for tumor driving mutations in a tumor, which can be focused on genes known to be related to the activated signaling pathway. Performing DNA and RNA sequencing simultaneously on the same sample is expected to provide highly complementary information. RNA sequencing data provide information on mutated genes that are actually expressed and likely to be functionally relevant and can be used to better link genomic mutations to abnormal STP activity. To apply this in the PACMAN study (PACMAN, 2021), STP activity analysis is performed retrospectively on samples across histologies of cancer which had been treated with targeted drugs guided by mutation analysis, resulting in targeted drug treatment for 19% of patients and a 7% increase in treatment response (Massard et al., 2017). Interim study results showed that addition of information on signaling pathway activity increased the percentage of breast and prostate cancers for which a targeted drug could be identified to nearly all cases (Martin et al., 2020).

### The Advantage of Assaying Low Input Analytes, Including Circulating Tumor Cells

Some clinical sample types are unavoidably associated with a minimal cancer cell content or low quality. Performing STP analysis on such samples in a standard manner tends to be associated with high technical noise (Inda et al., 2020). Obtaining tumor biopsies from difficult to access cancer types, such as lung cancer, or measuring pathway activity in circulating tumor cells (CTCs) may be clinically very relevant to obtain information on signaling pathway activity in the sample to decide on a targeted therapy choice. Furthermore, the pathways involved in epithelial to mesenchymal transition important for cell and CTC migration are reflected in our assay. Measuring targets in CTCs has high potential as a liquid biopsy of otherwise unattainable metastatic tumors and analyzed as single cells (van de Stolpe et al., 2011; van de Stolpe and den Toonder, 2014; Massagué and Obenauf, 2016).

**FIGURE 4:**
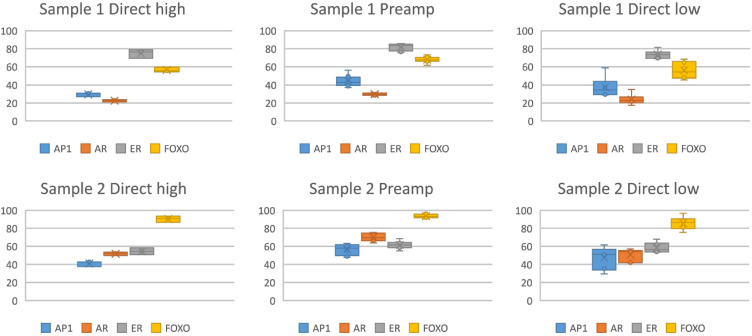
Measurement of activity of multiple signaling pathways simultaneously in a single small tissue sample, using RNA pre-amplification by multiplex PCR (96 primer sets) (Philips MPDx/OncoSignal, Eindhoven, Netherlands). Measurement of activity of the MAPK-AP1 (AP1), AR, ER, PI3K-FOXO (PI3K pathway activity is the inverse of measured FOXO activity) pathways in two independent formalin fixed paraffin embedded (FFPE) samples, activity score 0–100 on the *y*-axis. Left: direct high = pathway activity measurement without pre-amplification in a sufficiently large sample (*n* = 8), 1 ng per reaction, average mRNA qPCR Cq of 30. Middle: preamp = measurement in a very low RNA sample after pre-amplification, measured on sample (*n* = 6) diluted to average Cq of reference genes between 32 and 35.5. Right: direct low = measurement (*n* = 6) on a similar diluted sample without pre-amplification.

STP analysis of very small samples may be possible using either RNA sequencing or qPCR to measure the required mRNA levels. RNA sequencing on single cells has recently developed into a feasible technology (Hedlund and Deng, 2018; Hwang et al., 2018; Chen et al., 2019). Using the STP analysis technology, measurement of activity of the Wnt pathway on a single (circulating tumor) cell using RNA sequencing data has already been reported, underscoring the highly sensitive nature of the pathway analysis approach (van Strijp et al., 2017). Initial results using the qPCR-based STP analysis similarly indicated the feasibility of measuring pathway activity on only a few cells, using an adaptation of the protocol to include a pre-amplification method (Figure 4). Incorporating the RNA pre-amplification step is expected to enable performance of multiple STP analyses on a single CTC.

### Application of the mRNA-Based Assay Platform to Measure Activity of Signaling Pathways

Initial results have been published on a number of different cancer types using the above described assay platform, in which the potential of STP analysis was explored to provide insight in cancer pathophysiology and to predict prognosis and response to therapy (Verhaegh et al., 2014; van Boxtel et al., 2019; Inda et al., 2020; Sieuwerts et al., 2020; van Weelden et al., 2020). A few key results are summarized below, centered around the role of the different signaling pathways in the various cancer types.

The estrogen receptor (ER) pathway is a tumor driving signaling pathway in breast cancer, and can also be active in ovarian and endometrium cancer although its role in those malignancies is less clear. Adjuvant tamoxifen treatment of ER positive breast cancer patients was associated with significantly lower recurrence rate in patients with an ER pathway active primary tumor, compared to patients with an ER-inactive tumor (Verhaegh et al., 2014). Pathway analysis in three independent clinical studies (performed using both Affymetrix microarray- and qPCR-based pathway analysis) showed that in primary ER positive breast cancer patients, the ER pathway activity score predicted response to neoadjuvant treatment with an aromatase inhibitor (Inda et al., 2020). In ER positive breast cancer patients who developed metastatic disease and were subsequently treated with first line tamoxifen, an active ER pathway was associated with a better prognosis (Sieuwerts et al., 2020). In both ovarian cancer and endometrial cancer, loss of ER pathway activity was associated with higher grade cancer type and worse prognosis (van Lieshout et al., 2020; van Weelden et al., 2020). Results suggest that for all three cancer types, ER pathway activity reflects a more differentiated cancer type associated with a better prognosis, and measuring ER pathway activity may predict response to hormonal therapy in ER positive breast cancer.

The AR pathway is another important hormonal pathway and positive IHC staining has been described for different cancer types (Munoz et al., 2015). While, as expected, the AR pathway was universally measured as active in primary prostate cancer, AR pathway activity remained high in some castrate-resistant metastatic prostate cancers, possibly reflecting emergence of AR activating mutations (van de Stolpe et al., 2019a). In breast cancer patients, the highest average AR pathway activity score was found in the HER2 subtype (van de Stolpe et al., 2020). The ratio between AR and ER IHC staining has been suggested to be informative as to the functional role of the AR pathway in breast cancer (Rangel et al., 2018, 2020). STP analysis enabled calculation of an AR over ER pathway activity ratio instead of the AR over ER protein expression ratio, revealing a relatively low ratio in luminal A/B and a high ratio in higher grade HER2 and basal breast cancer subtypes. This is in line with reported AR/ER expression ratios per subtype, and suggestive of a potential benefit of anti-androgen therapy for breast cancer with a high AR/ER pathway activity ratio (van de Stolpe et al., 2020). In luminal breast cancer patients who had all developed metastatic disease and therefore constituted a bad prognosis patient subset, high AR pathway activity was associated with worse outcome (Sieuwerts et al., 2020). In advanced salivary duct cancer with positive AR IHC staining, the AR pathway activity score predicted response to androgen deprivation therapy (van Boxtel et al., 2019). Currently, a second independent clinical study is in progress aiming at confirmation of the predictive value of AR pathway activity for response to anti-androgen therapy.

The PI3K pathway is a major growth factor pathway, sometimes called a “survival” pathway, and the frequent pathway activity can be caused by for example mutations in the PIK3CA gene or amplification of the HER2 gene (van de Stolpe, 2019). In line with this, PI3K pathway activity was found to be associated with higher grade and worse prognosis in breast cancer, high grade serous ovarian cancer, prostate cancer, and esophageal cancer (Verhaegh et al., 2014; Creemers et al., 2018; van de Stolpe et al., 2019a; Sieuwerts et al., 2020; van Lieshout et al., 2020). Novel PI3K inhibitors including B591 and IBL-302 are in various stages of development.

Activity of developmental signaling pathways, such as the TGFβ, Hedgehog, Notch and Wnt pathways, is a typical characteristic of stem cells and was found in a variety of tumors including medulloblastomas and gliomas (Verhaegh et al., 2014; Holtzer et al., 2017; van de Stolpe et al., 2019a). Some of these pathways, such as the TGFβ pathway, may play either a tumor suppressive or tumor promoting role in cancer, depending on cellular context (Alinger et al., 2009; Katoh and Katoh, 2009; Massagué, 2012; Sundqvist et al., 2013; Liu et al., 2015; Aster et al., 2017). In breast cancer and colon cancer, high TGFβ pathway activity was associated with worse prognosis (Wesseling-Rozendaal et al., 2019a). In contrast, activity of the TGFβ pathway was frequently lost in advanced prostate cancer and in esophageal cancer, suggestive of a tumor suppressive role in the corresponding healthy tissue (Creemers et al., 2018; van de Stolpe et al., 2019a). Various anti-TGFβ small molecule inhibitors or antibodies are in various stages of development and include the compounds SAR439459 and galunisertib.

The Wnt pathway is well known for its tumor initiating role in colon adenoma and cancer, and Wnt pathway activity was confirmed in these tumors (Bienz and Clevers, 2000; Verhaegh et al., 2014). The role of Wnt pathway activity in some other cancers may be more complex (Kypta and Waxman, 2012; Schneider and Logan, 2018). In primary lower grade prostate cancer, Wnt pathway activity was associated with the TMPRSS2:ERG fusion protein, known to be able to activate the Wnt pathway, but in advanced disease Wnt pathway activity was frequently lost, suggesting a tumor-suppressive rather than tumor-promoting role under this condition (Wu et al., 2013). In primary breast cancer and in ovarian cancer, Wnt pathway activity seemed to be associated with less aggressive tumors (Sieuwerts et al., 2020; van Lieshout et al., 2020).

Notch pathway activity was associated with improved prognosis in T cell-acute lymphoblastic lymphoma (Canté-Barrett et al., 2020). Activity of the Hedgehog pathway was found in primary metastasized breast cancer and was associated with worse prognosis in luminal breast cancer patients who developed metastatic disease, and pathway activity was increased in higher compared to lower grade ovarian cancer (Beachy et al., 2018; Sieuwerts et al., 2020; van Lieshout et al., 2020). Signaling pathways frequently crosstalk to orchestrate specific cellular functions (Cantley et al., 2014). Simultaneous measurement of multiple signaling pathway activities on the same sample enabled exploration of cooperation between signaling pathways (Sieuwerts et al., 2020). Interaction between the SMAD2/3 transcription factor of the TGFβ pathway and the FOXO transcription factor (inversely related to PI3K pathway activity) has been described in detail, and can serve a tumor suppressive role (Massagué, 2012; Sundqvist et al., 2013). Activation of the PI3K pathway, reflected by loss of FOXO activity, is frequently associated with loss of TGFβ pathway activity (van Ooijen et al., 2018). In esophageal cancer treated with neoadjuvant chemoradiation, combined FOXO plus TGFβ pathway activity was associated with favorable outcome, while loss of FOXO and TGFβ pathway activity was associated with increased recurrence rate (Creemers et al., 2018). Cooperative activity of tumor driving signaling pathways may also be associated with resistance to a drug that targets a single pathway. This is explored in the PACMAN study, in which pathway analysis is performed on a variety of cancer types that were treated with targeted drugs (Martin et al., 2020). Multiple clinical studies are in progress to further explore the relation between single or combined signaling pathway activity and cancer progression, and to investigate clinical value of measuring signaling pathway activity to predict response and resistance to targeted treatment.

## Discussion: Future Applications With Therapy Selection

### Characterization of the Immune Microenvironment and Blood Samples

For many diseases, including cancer, the immune response is a major determinant of progression, response to therapy, and clinical outcome. Immunotherapy has emerged as a high potential curative treatment for many cancer types, and checkpoint inhibitor therapy is becoming standard of care for cancer types for a variety of tumors including melanoma, lung cancer, head and neck cancers (Havel et al., 2019). Various types of immunotherapy are available and being developed, mainly directed at breaking the immunotolerance against cancer or at inducing novel anti-cancer immunity by various vaccination approaches, and either as a monotherapy or in combination with radiotherapy, chemotherapy or targeted therapy (Emens and Middleton, 2015; Romero et al., 2016; Van Limbergen et al., 2017). While initially late-stage cancer has been the focus of immunotherapy, adjuvant use in an earlier clinical phase, and even in a neoadjuvant setting to exploit the tumor as a source of neo-antigen, is being explored (Moujaess et al., 2019). Aside from sporadic successes, such as microsatellite instability or mismatch repair-deficiency biomarkers to reliable predict checkpoint inhibitor response, challenges in predicting response to immunotherapy remain and currently used parameters such as tumor type, its neoantigen profile, histopathology of the tumor infiltrate (TIL) (e.g., inflammatory or immune excluded), and CD3+/CD8+ and PD1 and PD-L1 IHC do not perform sufficiently well (Chang et al., 2018; Hegde and Chen, 2020; Kennedy and Salama, 2020). As a consequence there is a high need for tests to improve response prediction and assessment, and also to predict who is at high risk for immune-mediated severe side effects (Duffy and Crown, 2019). Failure to successfully select responder patients for immunotherapy may ultimately endanger further clinical implementation of this high potential therapy.

The immune response is determined by coordinated activity of many immune cell types belonging to the innate and adaptive immune system. The functional state of immune cells is determined by coordinated activity of the same signal transduction pathways that have been described above as potential tumor driving signaling pathways (Newton and Dixit, 2012; Cantrell, 2015). Immune cells communicate through a variety of free (e.g., cytokines) or membrane-bound molecules that bind to specific cellular receptors to activate the signaling pathways that determine their function in mounting an immune response, such as T cell clonal expansion, cytotoxic functions and antigen presentation (Platanias, 2005; Li et al., 2007; Goodman et al., 2011; Lee et al., 2011; Han et al., 2012; Oh and Ghosh, 2013). Combined JAK-STAT1/2 and JAK-STAT3 pathway analysis in blood samples of patients with a variety of viral infections was shown to quantitatively measure the cellular immune response to viruses (Bouwman et al., 2020). With respect to cancer, measurement of STP activity in a tumor infiltrate (TIL), or possibly in blood samples, may be similarly informative on the immune-active versus immune-tolerant state of the immune response. Initial results suggest that in primary breast cancer the adaptive T cell response has already been switched off, while *in vitro* study results suggest that this may be mediated by soluble factors from cancer tissue (e.g., TGFβ) that reduce effector immune pathway activity (PI3K, JAK-STAT, NFκB pathways) and increase activity of the immune suppressive TGFβ pathway (Gu-Trantien et al., 2013; van de Stolpe et al., 2019b). Investigation of the value of STP analysis to predict response to checkpoint inhibitor immunotherapy is underway. New technologies are becoming available to separately analyze different cell types in cancer tissue, including immune cell subsets in the TIL (e.g., multiplex IHC, laser microdissection of selected regions for STP analysis). This may facilitate STP activity profiling in specific immune cell types in the TIL to investigate the relation with immunotherapy response (Gu-Trantien et al., 2018; Garaud et al., 2019; Solinas et al., 2019).

### Choosing a Targeted Drug for an Individual Patient Based on Identification of a Targetable Signal Transduction Pathway

**FIGURE 5:**
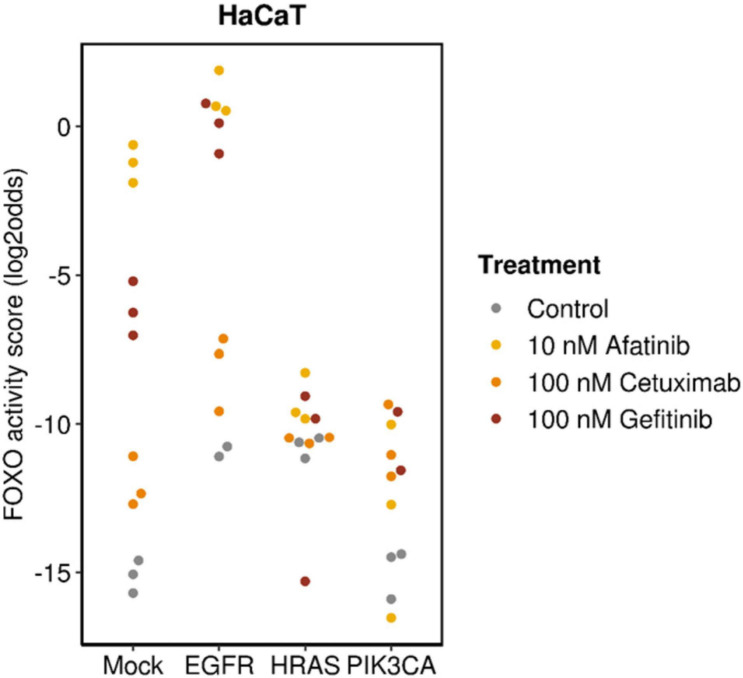
Analysis on data from GSE80667, described in Fertig et al. (2016). HaCaT premalignant keratinocyte cell line (mock), with overexpressed wild-type EGFR (EGFR),or activating mutation in HRAS (*HRAS*^V12D^) (HRAS) or PIK3CA (*PIK3CA*^H1047R^) (PIK3CA) genes; treated for 24 h with cetuximab, gefitinib, or afatinib. Measured FOXO activity score per analyzed sample, presented on a log2odds scale; PI3K pathway activity is the inverse of the FOXO activity score.

Choosing a targeted drug with the highest chance at clinical response requires characterization and consideration of a number of important factors. Since targeted drugs are specifically directed toward a component of a signaling pathway, a functionally active signaling pathway can be a prerequisite for response (Verhaegh et al., 2014; van Boxtel et al., 2019; Wesseling-Rozendaal et al., 2019b; Inda et al., 2020). In the presence of a pathway activating mutation in the gene for a signaling protein, the drug needs to target downstream of the activated protein in the signaling pathway to be effective. This is illustrated in Figure 5 in which anti-EGFR inhibitors (cetuximab, gefitinib, and afatinib) had been used to treat breast cancer cells containing mutations in genes coding for proteins that play a direct or indirect role (through crosstalk between pathways) in the PI3K pathway. All TKIs inhibited PI3K pathway activity in wild type cells and cells with overexpressed Epidermal Growth Factor Receptor (EGFR). Inhibition was maximal with the dual EGFR/HER2 inhibitor afatinib. Of the two EGFR TKI inhibitors, gefitinib was most effective, possibly because it targets EGFR intracellularly (Bardelli and Jänne, 2012). Only minor inhibitory effects were observed in *HRAS-* and *PIK3CA*-mutant cells because these mutations are located downstream of the TKI drug targets and therefore confer resistance.

A third factor to take into account in deciding on a targeted drug treatment is whether more than one tumor driving signaling pathway is active, potentially conferring drug resistance. This was a frequently encountered issue in the analyzed cancer types to date (Martin et al., 2020). Activity of the PI3K growth factor pathway in ER positive breast cancer was associated with primary resistance to endocrine therapy (Ciruelos Gil, 2014). Targeting of both pathways simultaneously may be tested to overcome resistance and improve clinical response. Inhibition of one signaling pathway may lead to activation of a resistance-conferring pathway. An *in vitro* example for the latter was identified in fulvestrant resistant breast cancer cell lines that unexpectedly had gained MAPK pathway activity in addition to loss of ER pathway activity (Wesseling-Rozendaal et al., 2019b).

### Dealing With Intra-Tumor Heterogeneity

Similar to heterogeneity with respect to mutations within a tumor, functional pathway activity levels may vary within a tumor. The amount of variation to expect probably depends on cancer type as well as the signaling pathway. In a clinical study on intra-tumor heterogeneity in breast cancer, ER pathway activity was the least variable and PI3K pathway activity the most variable within a tumor (van de Stolpe et al., 2018). Results of this study suggested that measuring pathway activity at two locations in a single primary breast cancer biopsy generally provided sufficient information on overall intra-tumor heterogeneity (van de Stolpe et al., 2018). Upon further confirmation, a repeat STP analysis on multiple parts of a biopsy may further underpin the choice for a targeted therapy. STP analysis is expected to also provide a novel tool, complementary to genome sequencing and methylation analysis, to investigate mechanisms of tumor evolution (Sottoriva et al., 2015; Sun et al., 2018; Roerink et al., 2018).

## Conclusion

Using the described STP analysis tests, activity of twelve relevant signaling pathways (PI3K, MAPK, JAK-STAT1/2, JAK-STAT3, NFκB, ER, AR, PR, Notch, TGFβ, Wnt and HH) can be measured simultaneously and quantitatively on individual patient cancer tissue samples from different cancer types. As illustrated above, this pathway analysis approach reveals commonalities with respect to pathway activities across different cancer types, potentially enabling an alternative cancer classification system based on signaling pathway activity profiles, complementary to the conventional classification based on the organ location. Developing a molecular fingerprint of tumors with respect to signaling pathway activities is expected to provide new insights into the pathophysiology of the many cancer types, including discovery of novel mechanisms for tumorigenesis and metastasis. STP analysis may also contribute to identification of novel drug targets and development of therapies that are more generalizable across cancer types including rare cancers.

Clinical diagnostic applications of STP analysis for prediction of prognosis and response to therapy, including identification of resistance pathways, are currently being further developed in clinical studies. Prospective clinical validation studies in various cancer types and basket studies are being initiated with clinical partners, predominantly making use of RT-qPCR-based STP analysis on FFPE tissue samples with considerations toward low quantity specimens including circulating tumor cells.

In the future, an important focus will lie on measuring the host immune response to cancer, both in blood as well as in cancer tissue samples. Taken together, measurement of STP activity in cancer, complementary to DNA mutation analysis, is expected to enable development of novel therapies, improve prediction of therapy response and resistance, and improve clinical outcome for a variety of tumor types and treatments, including targeted drugs and immunotherapy.

## Data Availability Statement

The raw data supporting the conclusions of this article will be made available by the authors upon request, without undue reservation.

## Ethics Statement

As far as data did not come from public data repositories, the studies involving human participants were reviewed and approved by local IRBs, and the patients/participants provided their written informed consent to participate.

## Author Contributions

HH and AS: conception and design, writing, review and comments. J-YB, CM, PP, MP, HP, DDR, NS, SS, and KW-G: review and comments. WV: modeling, writing, review and comments. All authors contributed to the article and approved the submitted version.

## Conflict of Interest

WV is an employee of Philips Research. J-YB, CM, PP, MP, HP, DDR, NS, SS, KW-G, and HH are on the advisory board of Philips MPDx.

## Footnotes

1 www.kegg.jp

2 www.wikipathways.org
